# Wounded Glioma Syndrome: A Case Report With Narrative Review

**DOI:** 10.7759/cureus.95494

**Published:** 2025-10-27

**Authors:** Fernando Roosemberg, Cristhian Ponce, Kevin Soto, Oswaldo Bolaños

**Affiliations:** 1 Critical Care, "Juan Tanca Marengo" National Oncological Institute, Guayaquil, ECU

**Keywords:** cerebral hemorrhage, distant wounded glioma syndrome, neurosurgery, peritumoral brain edema, postoperative complications, wounded glioma syndrome, glioma

## Abstract

Wounded glioma syndrome (WGS) is a rare but potentially fatal postoperative complication. It usually presents with cerebral edema and/or hemorrhage, which can develop even in regions of the brain distant from the surgical field, a phenomenon known as distant WGS.

A 72-year-old woman developed holocranial headache and progressive left hemiparesis. Brain magnetic resonance imaging (MRI) revealed an intra-axial lesion in the right hemisphere. She underwent right frontoparietal craniotomy with lobectomy and resection of the lesion. Her initial postoperative course was favorable. However, on the eighth day, she suffered acute neurological deterioration. Cranial computed tomography (CT) revealed severe cerebral edema and hemorrhagic foci in previously unoperated regions. The patient developed refractory intracranial hypertension that did not respond to intensive treatment (corticosteroids, deep sedation, and osmotherapy) and subsequently died. Histopathological analysis confirmed a diffuse grade 2 astrocytoma.

WGS poses a significant diagnostic and therapeutic challenge due to its low incidence and currently poorly understood pathophysiological behavior. Its unpredictable clinical course underscores the need for more comprehensive documentation and the development of standardized preventive protocols to mitigate the lethality of this entity.

## Introduction

Gliomas are primary tumors of the central nervous system arising from glial cells. They represent 30-40% of brain tumors and up to 80% of malignant intracranial tumors. Glioblastoma is the most aggressive variant with the poorest prognosis.

Wounded glioma syndrome (WGS) is a postoperative complication that may occur following biopsy or resection of gliomas. It is characterized by cerebral edema and hemorrhages that may occur even in regions not directly manipulated during surgery, referred to as distant WGS. Its clinical course is often abrupt and fatal. The pathophysiology remains poorly understood, and management lacks formal guidelines due to limited evidence, which is primarily based on case reports [1,2].

## Case presentation

**Figure 1:**
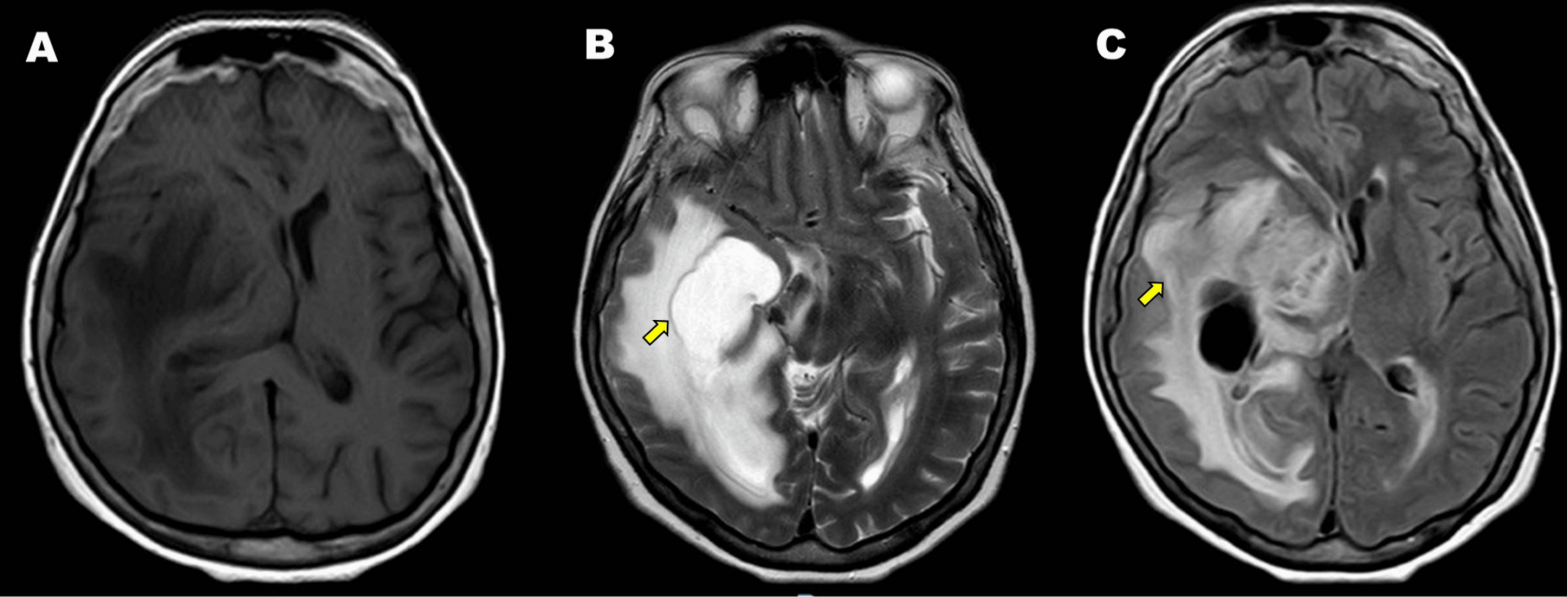
Brain MRI. Intra-axial lesion appearing hypointense on T1-weighted sequence (A), hyperintense on T2-weighted sequence (B), and hypointense on FLAIR sequence (arrow) (C), located in the right parieto-occipital region. The lesion measures approximately 4.8 cm in length and 2.6 cm in thickness, with contralateral midline shift and associated vasogenic edema (arrow) MRI: magnetic resonance imaging; FLAIR: fluid-attenuated inversion recovery

A 72-year-old mestizo woman with a history of hypertension had a baseline Karnofsky performance status of 70%. She presented with holocranial headache and progressive left brachio-crural hemiparesis. Brain magnetic resonance imaging (MRI) (Figure 1) revealed an intra-axial lesion with mild mass effect and vasogenic edema in the right temporoparietal and occipital regions.

**Figure 2:**
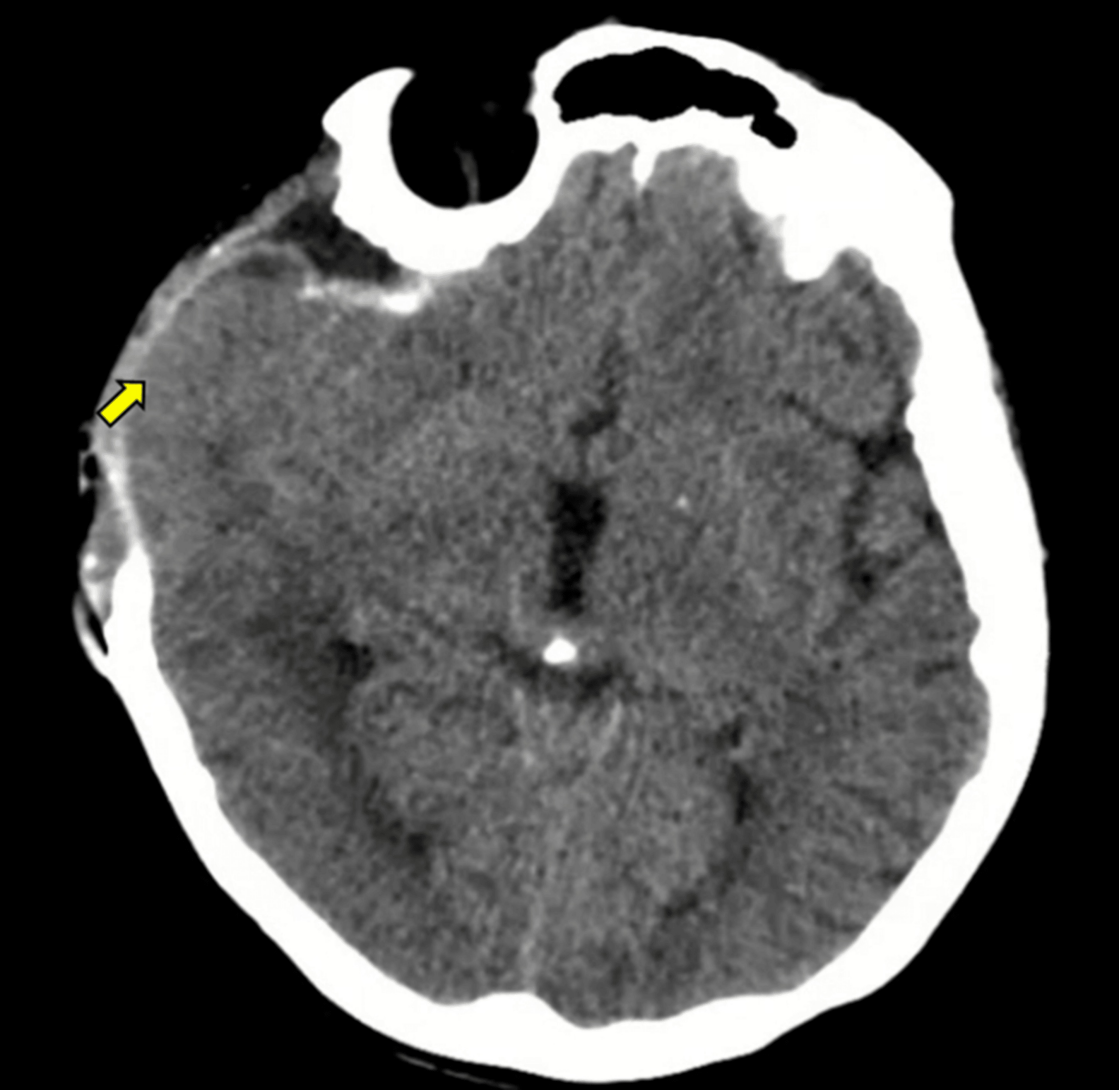
Non-contrast cranial CT scan. Postsurgical changes are observed in the cerebral parenchyma and bone tissue (arrow). No areas of hemorrhage or ischemia are identified CT: computed tomography

The patient underwent right frontoparietal craniotomy with decompression, right temporal lobectomy, cystic lesion resection, biopsy sampling, and duroplasty with dural substitute. The postoperative computed tomography (CT) scan showed no acute complications (Figure 2). Clinical evolution was favorable, and she was discharged on postoperative day 3. Pathology reported a World Health Organization (WHO) grade 2 diffuse astrocytoma.

**Figure 3:**
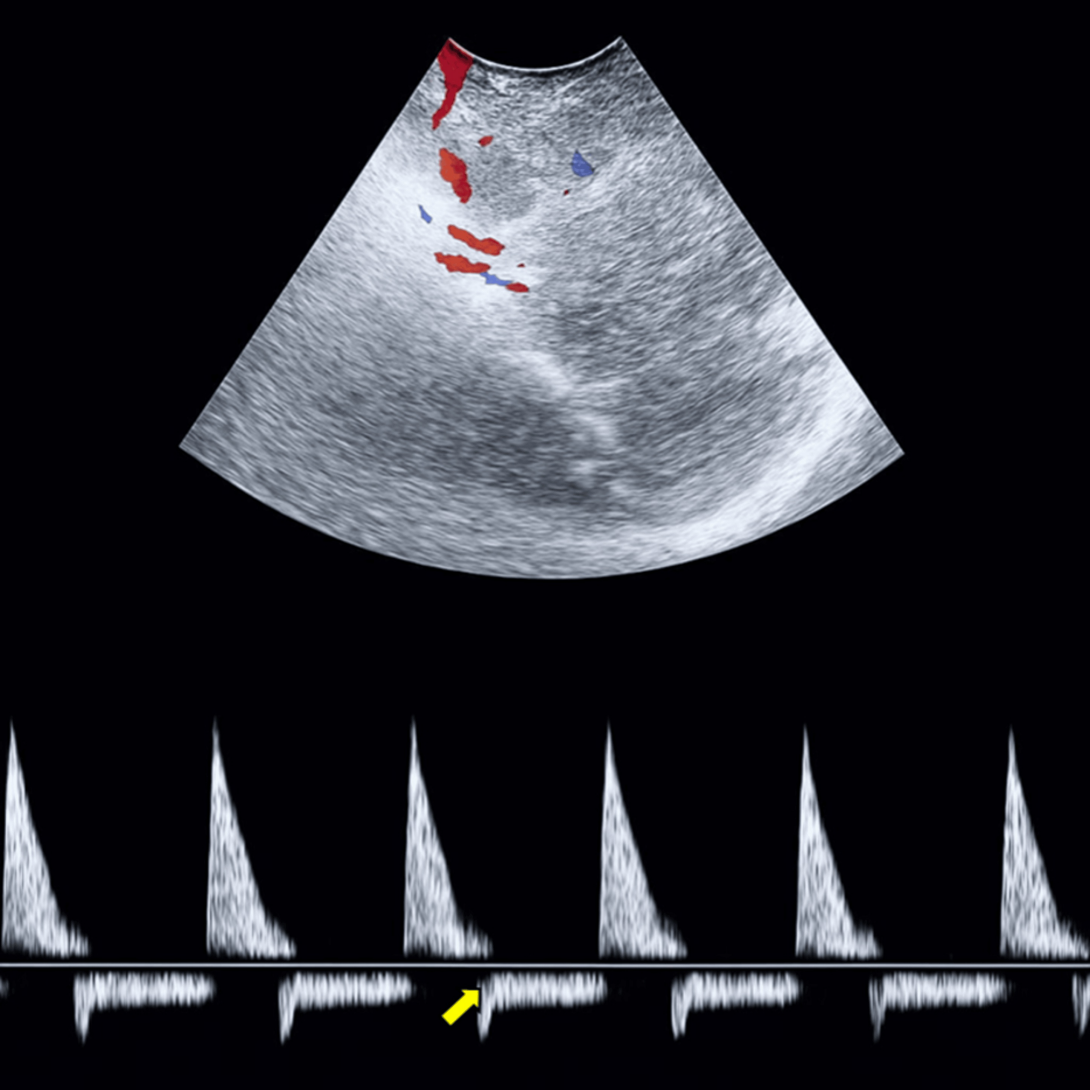
Transcranial Doppler of the right middle cerebral artery. A high-resistance pattern with reversed diastolic (arrow) flow is observed, suggestive of intracranial hypertension

On postoperative day 7, she was readmitted with altered consciousness (Glasgow Coma Scale 9/15: V4, M3, O2) [3], right hemiparesis, tachycardia, and arterial hypertension. She required intensive care unit admission, endotracheal intubation, and intensive neurological monitoring. Transcranial Doppler (TCD) revealed a high-resistance pattern with systolic spikes and reversed diastolic flow suggestive of severe intracranial hypertension in the right middle cerebral artery (Figure 3).

**Figure 4:**
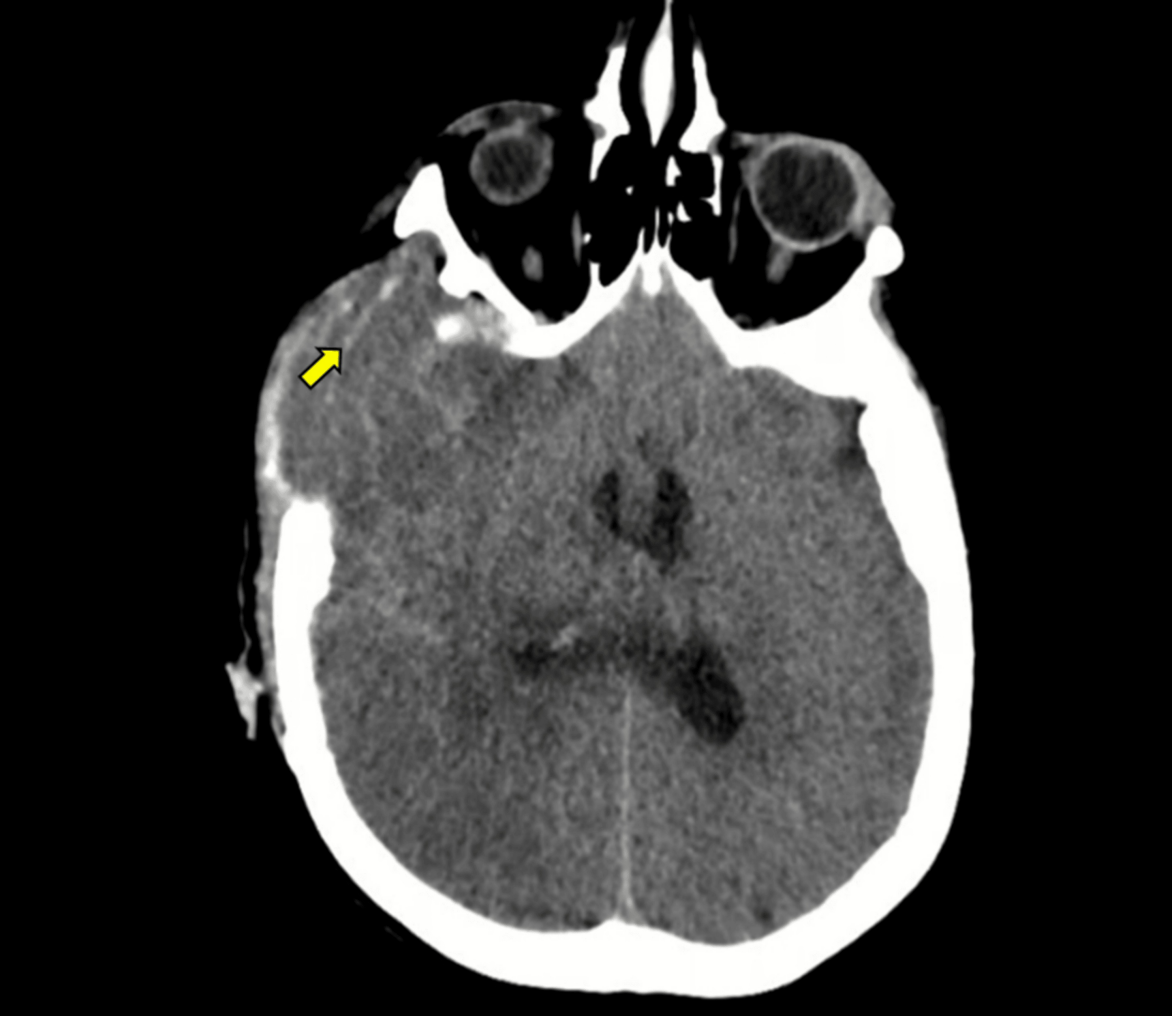
Non-contrast cranial CT scan. Diffuse cerebral edema is observed, along with scattered linear hyperdense areas in the right frontotemporal region, suspicious for hemorrhagic foci (arrow) CT: computed tomography

**Figure 5:**
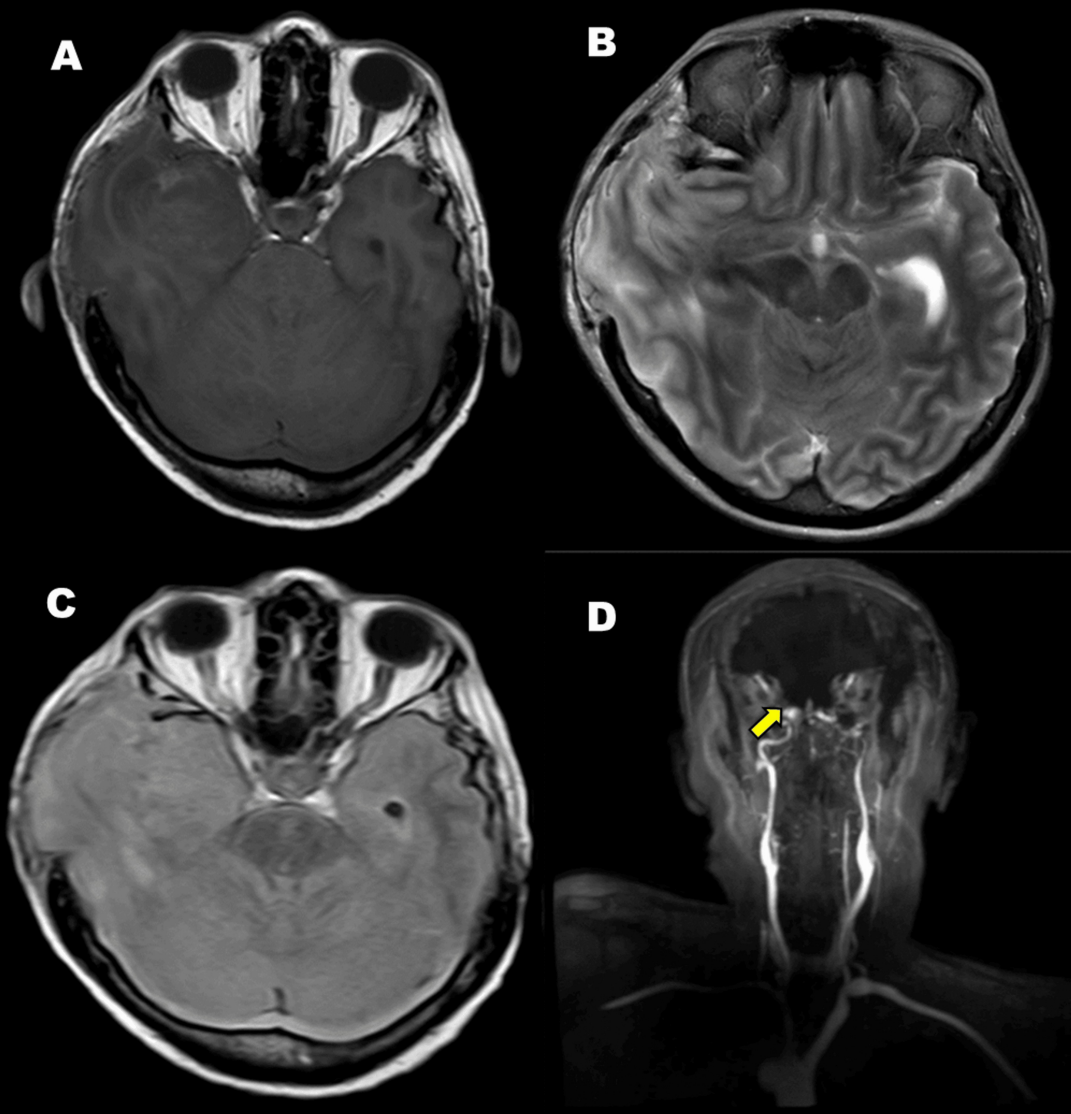
Brain MR angiography. Right frontotemporal craniectomy resulting in transcalvarial herniation. On T1 (A), T2 (B), and FLAIR (C) sequences, diffuse cerebral edema is noted, obliterating subarachnoid spaces in cortical sulci, fissures, and basal cisterns. Additionally, there is absence of vascular (arrow) flow in the vertebrobasilar system, circle of Willis arteries, and middle cerebral arteries, consistent with imaging signs of brain death (D) MR: magnetic resonance; FLAIR: fluid-attenuated inversion recovery

Subsequent CT scan demonstrated generalized cerebral edema and diffuse right frontotemporal hemorrhages (Figure 4). Within 24 hours, she developed bilateral fixed dilated pupils and absent cerebral blood flow on TCD. Brain MR angiography confirmed malignant cerebral edema, multifocal hemorrhages, and absence of cerebral perfusion consistent with brain death (Figure 5). Despite aggressive medical management with corticosteroids and osmotherapy, the patient died on the same day.

## Discussion

WGS is a rare but potentially devastating postoperative complication following tumor resection, especially when incomplete [1]. It has been reported after both craniotomy and stereotactic biopsy procedures, with perioperative complication rates between 6% and 12% [2].

The pathophysiology remains unclear. Resection of highly vascularized tissue may disrupt local hemodynamics, compromising regional cerebral blood flow and autoregulation, leading to peritumoral edema or hemorrhage [3,4]. Although bleeding typically occurs at the surgical site, in rare cases, it can occur in remote brain regions not directly manipulated during surgery, referred to as distant WGS [5].

Malignant cerebral edema is another life-threatening complication, often associated with refractory intracranial hypertension, brain herniation, and cerebral circulatory collapse [6]. Reported cases describe onset between the second and seventh postoperative days, coinciding with acute neurological deterioration and new deficits [7]. Incomplete resections with residual tumor volume greater than 70% have been associated with a higher risk [8]. Thus, maximal safe resection remains the preferred therapeutic strategy in glioblastoma to improve survival [9].

From a pathophysiological perspective, gliomas disrupt intracranial homeostasis through mass effect, requiring dynamic adjustments in cerebrospinal fluid (CSF) and cerebral blood flow (CBF) to maintain intracranial pressure. Abrupt tumor removal may induce compensatory disequilibrium, with increased flow through fragile residual vasculature, promoting bleeding. Anesthetic agents and intraoperative hyperventilation may further impair cerebral autoregulation and contribute to postoperative complications [10].

Distant WGS remains exceptionally rare. In a series of 4,992 intracranial procedures, only seven cases of remote hematomas were identified: three supratentorial after posterior fossa surgery and four contralateral to craniotomy [11]. Our case adds to this limited evidence, highlighting a fatal outcome following glioma resection. Tumor-related angiogenesis, overexpression of vascular endothelial growth factor (VEGF), and stress-induced release of inflammatory chemokines compromise the integrity of the blood-brain barrier [6]. In addition, the procoagulant properties of the tumor increase the risk of spontaneous hemorrhage, creating a vulnerable environment for postoperative events [12].

## Conclusions

Maximum safe resection remains the cornerstone of surgical treatment for glioblastoma, although it carries inherent risks. WGS and its variants are rare but potentially devastating complications, characterized by cerebral edema and hemorrhage. Given the limited evidence available, there are no standardized prevention protocols; however, strict blood pressure control, correction of coagulopathies, and prudent initiation of postoperative thromboprophylaxis may help mitigate the risk. The use of advanced technologies such as intraoperative MRI, neuronavigation, and fluorescence-guided surgery optimizes the extent of resection and improves safety, reducing recurrence and complications. From a clinical perspective, early detection of neurological abnormalities and timely intervention through a multidisciplinary approach are essential to improving prognosis. Looking ahead, multicenter data collection and systematic reporting are essential for establishing evidence-based preventive strategies and strengthening surgical safety in the management of glioblastoma.
